# G721-0282 Exerts Anxiolytic-Like Effects on Chronic Unpredictable Mild Stress in Mice Through Inhibition of Chitinase-3-Like 1-Mediated Neuroinflammation

**DOI:** 10.3389/fncel.2022.793835

**Published:** 2022-03-07

**Authors:** Hyeon Joo Ham, Yong Sun Lee, Hee Pom Lee, Young Wan Ham, Jaesuk Yun, Sang Bae Han, Jin Tae Hong

**Affiliations:** ^1^College of Pharmacy and Medical Research Center, Chungbuk National University, Cheongju, South Korea; ^2^Department of Chemistry, Utah Valley University, Orem, UT, United States

**Keywords:** chronic unpredictabe mild stress, neuroinflammation, chitinase-3-like 1, IGFBP3, microglia activation

## Abstract

Chronic stress is thought to be a major contributor to the onset of mental disorders such as anxiety disorders. Several studies have demonstrated a correlation between anxiety state and neuroinflammation, but the detailed mechanism is unclear. Chitinase-3-like 1 (CHI3L1) is expressed in several chronic inflammatorily damaged tissues and is well known to play a major role in mediating inflammatory responses. In the present study, we investigated the anxiolytic-like effect of N-Allyl-2-[(6-butyl-1,3-dimethyl-2,4-dioxo-1,2,3,4-tetrahydropyrido[2,3-d]pyrimidin-5-yl)sulfanyl]acetamide (G721-0282), an inhibitor of CHI3L1, on mice treated with chronic unpredictable mild stress (CUMS), as well as the mechanism of its action. We examined the anxiolytic-like effect of G721-0282 by conducting several behavioral tests with oral administration of G721-0282 to CUMS-treated BALB/c male mice. We found that administration of G721-0282 relieves CUMS-induced anxiety. Anxiolytic-like effects of G721-0282 have been shown to be associated with decreased expressions of CUMS-induced inflammatory proteins and cytokines in the hippocampus. The CUMS-elevated levels of CHI3L1 and IGFBP3 were inhibited by treatment with G721-0282 *in vivo* and *in vitro*. However, CHI3L1 deficiency abolished the anti-inflammatory effects of G721-0282 in microglial BV-2 cells. These results suggest that G721-0282 could lower CUMS-induced anxiety like behaviors by regulating IGFBP3-mediated neuroinflammation via inhibition of CHI3L1.

## Introduction

Anxiety disorders characterized by anxiety and fear are types of mental disorders that include generalized anxiety disorder, panic disorder, phobias, social anxiety disorder, obsessive-compulsive disorder (OCD), and post-traumatic stress disorder (PTSD) (Bandelow and Michaelis, 2015). According to World Health Organization (WHO) epidemiological data, more than one-third of the world’s population is affected by anxiety disorders, and thus anxiety disorders are considered the most prevalent mental illness (Gelfuso et al., 2014). Although there have been numerous studies of anxiety disorders, the pathogenesis of anxiety disorders has yet to be elucidated. Currently, drugs approved by the FDA for the treatment of anxiety disorders include selective serotonin reuptake inhibitor (SSRIs), serotonin norepinephrine reuptake inhibitors (SNRIs), tricyclic antidepressants (TCAs), benzodiazepines (BDZs), and monoamine oxidase inhibitors (MAOIs), which have serious side effects such as nausea, diarrhea, headache, insomnia, and sexual dysfunction, or have low therapeutic efficacy (Murrough et al., 2015). Therefore, there is a need to develop therapeutic agents for anxiety disorders.

Several studies have demonstrated a correlation between anxiety disorders and neuroinflammation (Salim et al., 2012). There is increasing evidence that pro-inflammatory and anti-inflammatory cytokines play an important role in anxiety disorders (Simen et al., 2006; Koo and Duman, 2009; Furtado and Katzman, 2015; Hou et al., 2017). The levels of pro-inflammatory cytokines such as tumor necrosis factor (TNF)-α and interferon (IFN)-γ are elevated in serum of patients suffering from anxiety disorder (Hou et al., 2017). NF-κB is well known as a transcription factor that regulates the expression of inflammatory genes (Kim et al., 2017). Inhibition of NF-κB signaling attenuated anxiety-related behaviors and NF-κB/p50 null mice showed decreased anxiety like behaviors (Koo et al., 2010). Additionally, several studies have found that Imipramine and Fluoxetine, widely used for the treatment of depressive and anxiety disorders, have NF-κB-inhibitory and anti-inflammatory effects (Troib and Azab, 2015). These data indicate that production of NF-κB-dependent neuroinflammatory cytokines could be significantly associated with the development of anxiety disorder.

Chitinase-3-like 1 (CHI3L1) is a secreted glycoprotein expressed in various cells including immune cells such as macrophages, neutrophils, and astrocyte (Lee et al., 2011; Choi et al., 2018). Growing evidence suggests that CHI3L1 could be a marker for inflammatory diseases (Bonneh-Barkay et al., 2010; Ahangari et al., 2015; Steponaitis et al., 2016; Querol-Vilaseca et al., 2017; Choi et al., 2018; Lee et al., 2019). CHI3L1 is up-regulated in inflammatory diseases such as rheumatoid arthritis, osteoarthritis, and asthma (Querol-Vilaseca et al., 2017). It is also reported that CHI3L1 is up-regulated in patients with chronic neurological disease such as Alzheimer’s disease, bipolar disorder, and schizophrenia (Bonneh-Barkay et al., 2010; Choi et al., 2018; Sahin et al., 2019). In our previous studies, we found that CHI3L1 deficiency attenuates inflammation-induced liver injury, and that administration of an inhibitor of CHI3L1 restores memory by alleviating neuroinflammation in an Alzheimer’s disease mouse model (Choi et al., 2018; Lee et al., 2019). It was reported that the expression level of proinflammatory cytokines such as TNF-α, IL-1β, and IL-6 was lower in CHI3L1 KO mice than in WT mice (Ahangari et al., 2015). Several studies have reported the association between CHI3L1 and NF-κB (Tang et al., 2013; Tran et al., 2014; Choi et al., 2018). Tran et al. (2014) have reported that the NF-κB signaling pathway is activated when CHI3L1 expression is increased. Choubey et al. (2019) reported that NF-κB is activated in restraint stress-induced anxiety status and that the degrees of anxiety and NF-κB activation are proportional. Zhu et al. (2018) also reported that CUMS dramatically activates NF-κB signaling, suggesting NF-κB plays a significant role in anxiety behavior. Thus, it is possible that CHI3L1 deficiency could ameliorate neuroinflammation and reduce anxiety.

Therefore, in the present study, we investigated whether N-Allyl-2-[(6-butyl-1,3-dimethyl-2,4-dioxo-1,2,3,4-tetrahydropyrido[2,3-d]pyrimidin-5-yl)sulfanyl]acetamide (G721-0282), an inhibitor of CHI3L1, could exert anti-anxiety effects through blockage of CHI3L1-mediated neuroinflammation.

## Materials and Methods

### Materials

**FIGURE 1:**
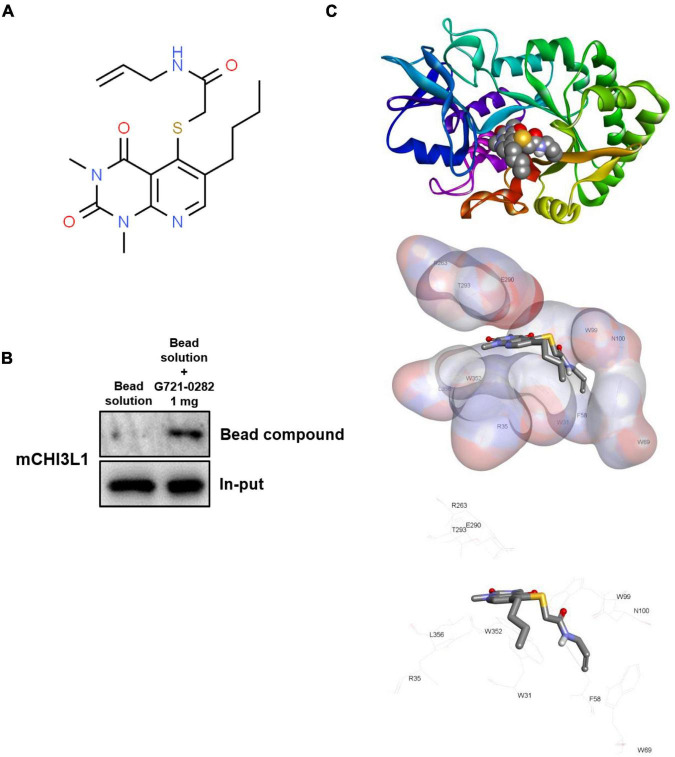
G721-0282 directly binds to CHI3L1. Chemical structure of G721-0282 **(A)**. Whole cell lysate of BV-2 cells were incubated with G721-0282-conjugated Sepharose 6B. After precipitation, the levels of bound CHI3L1 were monitored by Western blot analysis **(B)**. Docking model of G721-0282 and CHI3L1 **(C)**.

N-Allyl-2-[(6-butyl-1, 3-dimethyl-2, 4-dioxo-1, 2, 3, 4-tetrahydropyrido[2, 3-d]pyrimidin-5-yl)sulfanyl]acetamide (G721-0282) was obtained from ChemDiv, Inc. (San Diego, CA) (Figure 1A). The G721-0282 was dissolved in dimethyl sulfoxide (DMSO; final concentration of 100 mM) and stored at –20°C until use. The LPS was purchased from Sigma (serotype 0111:B4; Sigma, St. Louis, MO, United States). The LPS (final concentration of 1 mg/mL) was dissolved in PBS, and aliquots in PBS were stored at –20°C until use.

### Pull-Down Assay

G721-0282 was conjugated with Epoxy-activated Sepharose 6B (GE Healthcare Korea, Seoul, South Korea). Briefly, G721-0282 (1 mg) was dissolved in 1 mL of coupling buffer (0.1 M NaHCO_3_ and 0.5 M NaCl, pH 11.0). The Epoxy-activated Sepharose 6B beads (0.1 g) were swelled and washed in 1 mM HCl on a sintered glass filter, then washed with the coupling buffer. Epoxy-activated Sepharose 6B beads were added to the G721-0282-containing coupling buffer and rotated at 4°C overnight. The control unconjugated Sepharose 6B beads were prepared as described above in the absence of G721-0282. After washing, unoccupied binding sites were blocked with a blocking buffer (0.1 M Tris-HCl, pH 8.0) at room temperature for 3 h. The G721-0282-conjugated Sepharose 6B was washed with three cycles of alternating pH wash buffers (buffer 1: 0.1 M acetate and 0.5 M NaCl, pH 4.0; buffer 2: 0.1 M Tris-HCl and 0.5 M NaCl, pH 8.0). G721-0282-conjugated beads were then equilibrated with a binding buffer (0.05 M Tris-HCl and 0.15 M NaCl, pH 7.5). To demonstrate binding of G721-0282 and CHI3L1, the CHI3L1 protein was overexpressed by transfection with CHI3L1 DNA. BV-2 cells were transfected with CHI3L1 DNA (700 ng/per well of a six-well plate) using Lipofectamine^®^ 3000 (Invitrogen, Waltham, MA, United States) in Opti-MEM, following the manufacturer’s protocol. The BV-2 cell lysate (1 mg of protein) was mixed with G721-0282-conjugated Sepharose 6B or unconjugated Sepharose 6B and incubated at 4°C overnight. The beads were then washed three times with TBST. The bound proteins were eluted with sodium dodecyl sulfate (SDS) loading buffer and were separated using SDS/polyacrylamide gel electrophoresis, followed by immunoblotting with antibodies against CHI3L1 (1:1,000; Abcam, Inc., Cambridge, United Kingdom).

### Docking Experiment

A docking study of G721-0282 with CHI3L1 was performed using Autodock VINA. The three-dimensional structures of CHI3L1 (PDB: 1NWR) were retrieved from the Protein Data Bank and further prepared using AutodockTools. The grid box was centered on the CHI3L1 monomer, and the size of the grid box was adjusted to include the whole monomer. Docking experiments were performed at various default exhaustiveness values: 16, 24, 32, 40, and 60. Molecular graphics for the best binding model were generated using the Discovery Studio Visualizer 2.0.

### Animals and Treatment

**FIGURE 2:**
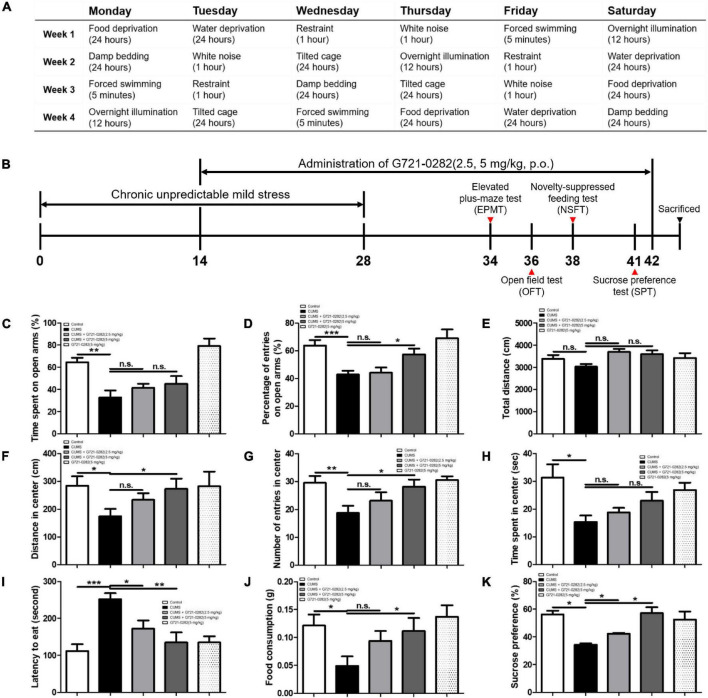
Anxiolytic-like effect of the G721-0282 on CUMS-induced anxiety behaviors in BALB/c mice. A table have been described that the daily CUMS schedule and the stressors used in the CUMS procedures **(A)**. A timeline have been described that demonstrate the administration of G721-0282 and the serial assessment of anxiety in BALB/c mice **(B)**. To investigate the anxiolytic-like effect of G721-0282, we carried out the EPMT **(C,D)**, OFT **(E–H)**, NSFT **(I,J)**, and SPT **(K)**. Anxiety level in BALB/c mice was determined by time spent on open arms (**C**, %) and the number of entries on open arms (**D**, %) in the EPMT, by distance in center (**F**, cm), the number of entries in center (**G**, counts), and time spent in center (**H**, s) in OFT, by latencies to eat (**I**, s) and food consumption (**J**, g) in NSFT, and by sucrose preferences (**K**, %) in SPT. *, Significantly different between two groups (*p* < 0.05). ^**^, Significantly different between two groups (*p* < 0.01). ^***^, Significantly different between two groups (*p* < 0.001).

Eight-week-old male BALB/c mice were maintained and handled in accordance with the humane animal care and use guidelines of the Ministry of Food and Drug Safety. BALB/c mice were purchased from Daehan Biolink (Chungcheongbuk-do, South Korea) and were given 7 days of adaptation time before chronic unpredictable mild stress (CUMS) schedule. The mice were randomly divided into five groups: (I) the control vehicle-treated group without CUMS treatment; (II) the vehicle-treated group with CUMS treatment; (III) the G721-0282 (2.5 mg/kg)-treated group with CUMS treatment, (IV) the G721-0282 (5 mg/kg)-treated group with CUMS treatment; and (V) the G721-0282 (5 mg/kg)-treated group without CUMS treatment. Groups II–IV were exposed to a variable sequence of mild and unpredictable stressors for 4 weeks in a procedure that produces anxiety-like behavioral changes. The CUMS schedule was modified from Xu et al. (2018) (Figure 2A). The groups were exposed to the folowing stressors randomly and continuously: (1) white noise, (2) forced swimming (5 min), (3) food or water deprivation for 24 h, (4) dirty cage (200 mL water in 100 g bedding), (5) 45°cage tilt, (6) overnight illumination, and (7) restraint for 1 h. The G721-0282 was given by oral gavage to groups III and IV twice per week for 4 weeks (Figure 2B). Groups I and II were alternatively given an equal volume of vehicle. The behavioral tests measuring the degree of anxiety were assessed using the elevated plus-maze test (EPMT), the open field test (OFT), the novelty suppressed feeding test (NSFT), and the sucrose preference test (SPT). Mice were sacrificed after behavioral tests by CO_2_ asphyxiation.

### Elevated Plus-Maze Test

Elevated plus-maze apparatus consisting of two perpendicular open (30 × 5 cm) and two closed arms (30 × 5 cm) with 15-cm-high walls, extending from the central platform (5 × 5 cm), was used. The maze was elevated to a height of 38 cm above the floor level. Elevated plus-maze testing was carried out by the SMART-CS (Panlab, Barcelona, Spain) program and equipment. A minute for adaptation was given prior to testing, and a standard 5-min test was employed for each mouse. All experimental sessions were recorded with a video camera mounted vertically above the maze. The activity in the open arm was evaluated as: (1) time spent on the open arms relative to the total time spent in the plus maze, expressed as a percentage; (2) the number of entries to both arms, also expressed as a percentage. The activity in the open arm was monitored by a camera above the center of the apparatus connected to a SMART-LD program (Panlab, Barcelona, Spain).

### Open Field Test

A mouse was placed in the corner of a black box (dimensions: 60 × 60 × 45 cm) and its travel pattern was monitored and recorded for 10 min using the SMART-LD program (Panlab, Barcelona, Spain). The locomotor activity was evaluated as: (1) time spent in the center; (2) distance in the center; (3) the number of entries in the center.

### Novelty Suppressed-Feeding Test

After fasting for 24 h, a mouse was placed in the corner of the black box used in OFT (60 × 60 × 45 cm) with a few pallets in the center. For 5 min, the latency to start eating the food and the food consumption were measured.

### Sucrose Preference Test

For the SPT, the mice were simultaneously exposed to tap water and 1% sucrose solution for 48 h, followed by 24 h of water and food deprivation. They were then exposed to two identical bottles for 1 h, one filled with the 1% sucrose solution and the other filled with tap water. The sucrose and water consumption were determined by measuring the volume of liquid consumed. The sucrose preference is defined as the ratio of the volume of sucrose solution consumed to the total volume of sucrose solution and water consumed over an hour.

### Collection and Preservation of Brain Tissues

After behavioral tests, mice were perfused with PBS with heparin under inhaled CO_2_ anesthetization. The brains were immediately removed from the skulls, after which only the hippocampus region was isolated and stored at –80°C until biochemical analysis.

### Western Blot Analysis

Homogenized hippocampus tissues and cells were lysed by protein extraction solution (PRO-PREP, iNtRON, Sungnam, South Korea) and the total protein concentration was determined using the Bradford reagent (Bio-Rad, Hercules, CA, United States). 80 μg of extracted protein were separated by SDS/PAGE and transferred to Immobilon^^®^^ PVDF membranes (Millipore, Bedford, MA, United States). The membrane was blocked with 5% BSA for 1 h at room temperature, followed by incubation with specific primary antibodies overnight at 4°C. The membranes were washed with Tris-buffered saline containing 0.05% Tween-20 (TBST) and incubated with diluted horseradish peroxidase-conjugated secondary antibodies for 1 h at room temperature. After washes, binding of antibodies to the PVDF membrane was detected using the Immobilon Western Chemilum HRP substrate (Millipore, Bedford, MA, United States). The band intensities were measured using the Fusion FX 7 image acquisition system (Vilber Lourmat, Eberhardzell, Germany) and quantified using Image J software. To detect target proteins, specific antibodies against CHI3L1, iNOS, IBA-1, and GFAP (1:1,000; Abcam, Inc., Cambridge, United Kingdom), COX-2 (1:1,000; Novus Biologicals, Inc., CO, United States), NF-κB inhibitor (IκB)α, p-IκBα, p65, and p50 (1:000; Cell signaling Technology, Inc., MA, United States), IGFBP3 (1:1,000; Abnova, Inc., Taipei, Taiwan), BDNF (1:1,000; Arigo Biolaboratories, Inc., Taiwan), and β-actin (1:200; Santa Cruz Biotechnology Inc., Santa Cruz, CA, United States) were used. The blots were then incubated with the corresponding conjugated secondary antibodies such as anti-mouse, anti-rabbit, and anti-goat purchased from Santa Cruz Biotechnology Inc. (Santa Cruz, CA, United States).

### RNA Isolation and Quantitative Real-Time RT-PCR

Total RNA was extracted using RiboEX (Geneall biotechnology, Seoul, South Korea) from hippocampus tissue and BV-2 cells, and cDNA was synthesized using High-Capacity cDNA Reverse Transcription kit (Thermo Scientific, Waltham, MA, United States). Quantitative real-time PCR was performed on a 7,500 real-time PCR system (Applied Biosystems, Foster City, CA, United States) for custom-designed primers and β-actin was used for house-keeping control using HiPi Real-Time PCR SYBR green master mix (ELPIS biotech, Daejeon, South Korea). Cycling conditions consisted of an initial denaturation step of 3 min at 94°C, a denaturation step of 30 s at 94°C, an annealing step of 30 s at 60°C, and an extension step of 1 min at 72°C followed by 40 cycles. The values obtained for the target gene expression were normalized to β-actin and quantified relative to the expression in control samples. Each sample was run with a primer pair of the same sequence as shown in Supplementary Table 1.

### Immunohistochemistry

The brain fixed in a 10% formalin solution was embedded in paraffin wax, and then the brain was cut into sections 5-μm-thick slices. Immunohistochemistry was performed as described previously (Ham et al., 2019b). To detect target proteins, specific antibodies against IBA-1 (1:500; Wako Pure Chemical Industries, Ltd., Osaka, Japan) was used. Brain sections were visualized by a chromogen diaminobenzidine (Vector Laboratories, Burlingame, CA, United States). Finally, brain sections were mounted with mounting medium Cytoseal XYL (Thermo Fisher Scientific, Waltham, MA, United States), and evaluated on a light microscope (Microscope Axio Imager. A2; Carl Zeiss, Oberkochen, Germany; × 50 and × 200).

### BV-2 Microglial Cell Culture

Microglial BV-2 cells were maintained with serum-supplemented culture media of DMEM supplemented with FBS (10%) and antibiotics (100 units/mL). The microglial BV-2 was incubated in the culture medium in a humidified incubator at 37°C and 5% CO_2_. The cultured cells were treated with several concentrations (5, 10, 20 μM) of G721-0282 and LPS (1 μg/mL). The cells were harvested after 24 h.

### Cell Viability Assay

BV-2 cells were plated in 96-well plates and subsequently treated with G721-0282 (5, 10, 20 μM) for 24 h. After treatment, cell viability was measured by MTT [3-(4, 5-Dimethylthiazol-2-yl)-2, 5-Diphenyltetrazolium Bromide] solution (Sigma Aldrich, St. Louis, MO). MTT solution having a volume of 1/10 of the culture medium was added to each well, and the mixture was incubated for 2 h in a CO_2_ incubator and then removed. After removing the mixture from the cells, DMSO of the same volume as that of the medium was added, and then the absorbance of each well was read at a wavelength of 570 nm using a microplate absorbance reader.

### Nitric Oxide Assay

BV-2 cells were grown in 96-well plates and then incubated with or without LPS (1 μg/mL) in the absence or presence of various concentrations of G721-0282 (5, 10, 20 μM) for 24 h. The nitrite concentration in the supernatant was assessed by NO detection kit (Intron Biotechnology, Kyungki-do, South Korea). The absorbance at 520 nm was measured in a microplate absorbance reader, and a series of known concentrations of sodium nitrite was used as a standard.

### Transfection

BV-2 cells were transiently transfected with siRNA of the target gene or negative control (NC) (20nM/well/6-well plate) using the Lipofectamine^®^ RNAiMAX transfection reagent in OPTI-MEM, according to the manufacturer’s specification (Invitrogen, Waltham, MA, United States). Negative control, CHI3L1, IGFBP3 siRNA were purchased from OriGene Technologies, Inc. (Rockville, MD, United States).

### Gene Network Analyses

The gene network of CHI3L1 was analyzed using the web-based analysis tools, STRING (string-db.org) and GeneMANIA,^1^ based on the publicly available biological datasets in terms of co-expression, co-localization, genetic interactions, pathway, physical interactions, predicted interactions, and shared protein domains. The gene network is automatically analyzed by gene-ontology base weighting methods.

### Measurement of Serum Corticosterone Level

Serum corticosterone (CORT) levels were determined using each specific CORT enzyme-linked immunosorbent assay (ELISA) Kit (MyBioSource, San Diego, CA, United States), according to the manufacturer’s instructions. The color development was stopped and the intensity of the color was measured at 450 nm in a microplate absorbance reader.

### Statistical Analysis

The data were statistically analyzed using the GraphPad Prism software (Version 4.03; GraphPad software, Inc., San Diego, CA, United States). Data are presented as mean ± S.E.M. The group differences in all data were assessed by one-way analysis of variance (ANOVA) followed by the Tukey multiple comparison test. A value of *p* < 0.05 was considered statistically significant. *, Significantly different between two groups (*p* < 0.05). ^**^, Significantly different between two groups (*p* < 0.01). ^***^, Significantly different between two groups (*p* < 0.001).

## Results

### G721-0282 Directly Binds to Chitinase-3-Like 1

We screened 14 million compounds against the ChemBridge corporation (San Diego, CA, United States). Among them, we selected the G721-0282 compound since implementing structure-based virtual screening and computational docking study using the Glide software (Schrödinger Inc., NY, United States) showed strong binding to CHI3L1 (–7.2 kcal/mol). Moreover, G721-0282 has good drug likeness without toxicities predicted with preADMET^2^ and StarDrop^3^ (Supplementary Table 2).

Since G721-0282 targets CHI3L1 and exhibits a CHI3L1 inhibitory effect, we investigated whether G721-0282 could interact physically with CHI3L1 through a pull-down assay and a docking experiment. CHI3L1 level was much higher in G721-0282-Sepharose 6B beads, suggesting that G721-0282 binds to CHI3L1 (Figure 1B). To investigate the binding site of G721-0282 to CHI3L1, a virtual docking analysis between G721-0282 and CHI3L1 was performed (Figure 1C). The binding study showed a surface rendering of CHI3L1 with G721-0282 and that G721-0282 binds directly to several amino acids in CHI3L1 (Trp31, Arg35, Phe58, Trp69, Trp99, Asn100, Arg263, E290, Thr293, Trp352, and Leu356; binding affinity for best binding interaction = –7.2 kcal/mol).

### G721-0282 Alleviates Anxiety Induced by Chronic Unpredictable Mild Stress

To investigate the anxiolytic-like effect of G721-0282, the degree of anxiety was assessed through elevated plus maze test (EPMT), open field test (OFT), novelty suppressed feeding test (NSFT), and sucrose preference test (SPT).

In the EPMT, the longer the mouse stays in the open arms and the greater the number of times it enters the open space, the less anxious it is (Figures 2C,D). Average percentage of time spent and percentage of number of entries in open arms were significantly reduced in the CUMS-vehicle group. On the other hand, in the groups treated with G721-0282, the percentage increased in a concentration dependent manner treated with G721-0282 [*n* = 6–8; percentage of time spent: *F*_(4, 26)_ = 11.20, *p* < 0.0001; percentage of number of number of entries: *F*_(4, 28)_ = 7.172, *p* = 0.0004].

In the OFT, the more the mouse travels to the center of box, the more it enters the center, and the longer it stays in the center, the less anxious it is. There was no difference in the total distance of all groups [*n* = 6–8; *F*_(4, 35)_ = 2.165, *p* = 0.0934] (Figure 2E). The distance in the center was significantly lower in the CUMS-vehicle group. However, in the groups treated with G721-0282, the distance was greater in the 5 mg/kg group [*n* = 6–8; *F*_(4, 31)_ = 1.816, *p* = 0.1509] (Figure 2F). The number of entries into the center was remarkedly reduced in the CUMS-vehicle group. In the groups treated with G721-0282, the number was greater in the 5 mg/kg group [*n* = 6–8; *F*_(4, 35)_ = 4.164, *p* = 0.0073] (Figure 2G). Time in the center was significantly lower in the CUMS-vehicle group. However, in the groups treated with G721-0282, the time was increased, although not statistically significant, in a concentration dependent manner [*n* = 6–8; *F*_(4, 29)_ = 3.477, *p* = 0.0195] (Figure 2H).

In the NSFT, the longer the latency to eat foods located in the middle of the box and the less the amount of food taken, the higher the anxiety level of the mouse. The latency was elevated in the CUMS-vehicle group than that in control group without CUMS, however, the letency in the groups treated with G721-0282 was decreased [*n* = 6–8; *F*_(4, 35)_ = 7.647, *p* = 0.0002] (Figure 2I). The amount of food consumed was significantly lower in CUMS compared to that in control group without CUMS, whereas, in the groups treated with G721-0282, the amount was increased [*n* = 6–8; *F*_(4, 33)_ = 3.252, *p* = 0.0236] (Figure 2J).

In the SPT, the more stressed the mouse, the less likely it is to consume sucrose solution. Sucrose preference was decreased in CUMS-vehicle group compared to that in control group without CUMS. In the groups treated with G721-0282, the amount was significantly greater than that in CUMS-vehicle group [*n* = 6–8; *F*_(4, 5)_ = 7.925, *p* = 0.0216] (Figure 2K).

Taken together, these results regarding differences in behavior indicate that increased anxiety due to CUMS is alleviated by the administration of G721-0282.

### G721-0282 Inhibits Neuroinflammation *in vivo* and *in vitro*

**FIGURE 3:**
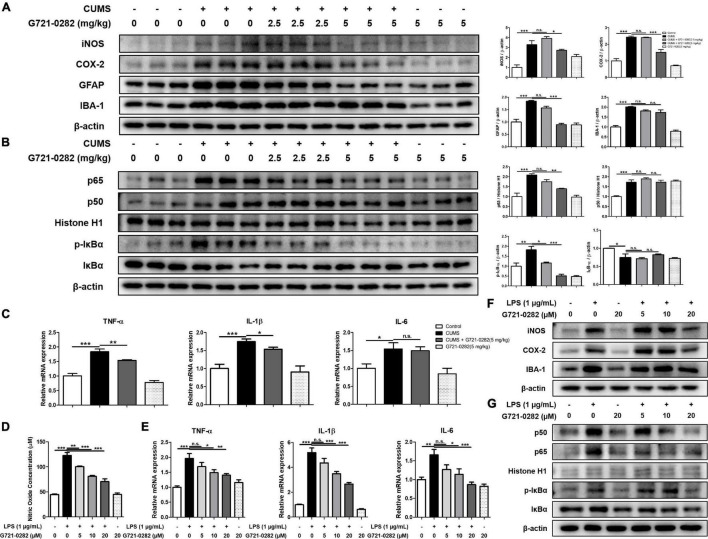
Inhibitory effect of G721-0282 on CUMS and LPS-induced neuroinflammation. The expression of iNOS, COX-2, GFAP and IBA-1 were detected by Western blot in the mice brain **(A)**. The translocation level of p65 and p50 (Nuclear) and the phosphorylation level of IκB were detected by Western blot in mice brain **(B)**. The loading control, β-actin, was reused in **(A,B)** and Figure 5D, because **(A,B)** and Figure 5D were obtained from the same amount of the same samples. The mRNA expression level of pro-inflammatory cytokines (TNF-α, IL-1β, and IL-6) in the mice brain hippocampus site were assessed by qRT-PCR **(C)**. The effect of G721-0282 on Nitric Oxide production in BV-2 cells were measured by Nitric Oxide assay **(D)**. The mRNA expression level of pro-inflammatory cytokines (TNF-α, IL-1β, and IL-6) in BV-2 cells treated with G721-0282 were assessed by qRT-PCR **(E)**. The expression of iNOS, COX-2, and IBA-1 were detected by Western blot in BV-2 cells treated with G721-0282 **(F)**. Phosphorylation of IκB, and p50 and p65 (Nuclear) translocation were detected by Western blot in BV-2 cells treated with G721-0282 **(G)**. *, Significantly different between two groups (*p* < 0.05). ^**^, Significantly different between two groups (*p* < 0.01). ^***^, Significantly different between two groups (*p* < 0.001).

We performed Western blotting and qRT-PCR to determine how chronic stress affects the inflammatory responses in the hippocampus and how G721-0282 would affect these responses. We investigated the levels of inflammatory protein, iNOS and COX-2, and the level of GFAP and IBA-1, the markers of reactive astrocyte and activated microglia, respectively, in the hippocampus of mice, by Western blotting. The levels of iNOS, COX-2, GFAP, and IBA-1 were increased in the CUMS-treated group and their expression levels decreased in a concentration-dependent manner upon treatment with G721-0282 (Figure 3A). Because NF-κB signaling is known to be highly correlated with inflammatory responses, we examined the effect of CUMS on NF-κB signaling and the effect of G721-0282 treatment by Western blotting. The translocation level of p65 and p50 in the nucleus, and the level of p-IκBα in the hippocampus of the CUMS-treated group were significantly increased, suggesting that CUMS could activated NF-κB signaling (Figure 3B). However, the translocation levels of p65, p50, and p-IκBα were lower in the group treated with CUMS and G721-0282 than in the group treated with CUMS alone. The production of pro-inflammatory cytokines in the CNS is closely related to the generation of neuroinflammation. qRT-PCR was performed to quantitatively analyze the expression of pro-inflammatory cytokines in the mouse hippocampus. In the hippocampus of the CUMS-treated group, the expression of proinflammatory cytokines such as TNF-α, IL-1β, and IL-6 was significantly elevated compared to the control group and showed a lower expression level in the CUMS and G721-0282-treated groups [*n* = 6–8; TNF-α: *F*_(3, 29)_ = 41.25, *p* < 0.0001; IL-1β: *F*_(3, 29)_ = 12.64, *p* < 0.0001; IL-6: *F*_(3, 29)_ = 5.855, *p* = 0.0030] (Figure 3C). Thus, chronic stress induces neuroinflammation by activating NF-κB signaling, while administration of G721-0282 mitigates neuroinflammation by inactivating NF-κB signaling.

Inhibitory effects of G721-0282 on neuroinflammation in microglia were determined in terms of nitric oxide concentration and expression levels of inflammatory-related proteins and cytokines. Immunohistochemistry was performed to determine how microglia are affected by CUMS and G721-0282 in the hippocampus of mice (Supplementary Figure 1). The number of IBA-1 reactive cells was increased by CUMS induction, and decreased by G721-0282 treatment. The effect of G721-0282 on cell viability in BV-2 cells was first determined by MTT assay (Supplementary Figure 2). There was no significant change in cell viability in the G721-0282 (5, 10, 20 μM)-treated groups compared to the control group. Thus, G721-0282 does not appear toxic to BV-2 cells up to 20 μM. Elevated nitric oxide concentration by LPS was significantly reduced by G721-0282 treatment in a concentration-dependent manner [*n* = 8; *F*_(5, 42)_ = 67.74, *p* < 0.0001] (Figure 3D). LPS-induced levels of pro-inflammatory cytokines were significantly decreased in a concentration-dependent manner in G721-0282-treated groups [*n* = 8; TNF-α: *F*_(5, 42)_ = 10.79, *p* < 0.0001; IL-1β: *F*_(5, 42)_ = 64.29, *p* < 0.0001; IL-6: *F*_(5, 42)_ = 8.075, *p* < 0.0001] (Figure 3E). We observed that the expression of inflammatory markers iNOS, COX-2, and IBA-1 was higher in the LPS-treated group and lower in the G721-0282-treated groups (Figure 3F). The increased translocation levels of p65, p50, and p-IκBα were reduced in the G721-0282-treated groups (Figure 3G).

### The Reversal of Inhibitory Effects of G721-0282 on Neuroinflammation in a Chitinase-3-Like 1-Deficient Environment

**FIGURE 4:**
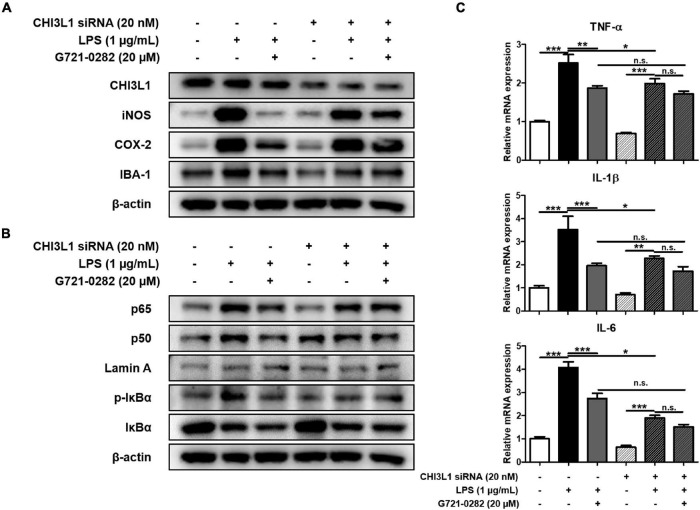
Offset of effects of G721-0282 on neuroinflammation by CHI3L1 siRNA in BV-2 cells. The expression of iNOS, COX-2, and IBA-1 were detected by Western blot in BV-2 cells treated with siCHI3L1 and G721-0282 **(A)**. Phosphorylation of IκB, and p50 and p65 (Nuclear) translocation were detected by Western blot in BV-2 cells treated with siCHI3L1 and G721-0282 **(B)**. The loading control, β-actin, was reused in **(A,B)**, because **(A,B)** were obtained from the same amount of the same samples. The mRNA expression level of pro-inflammatory cytokines (TNF-α, IL-1β, and IL-6) in BV-2 cells treated with siCHI3L1 and G721-0282 were assessed by qRT-PCR **(C)**. *, Significantly different between two groups (*p* < 0.05). ^**^, Significantly different between two groups (*p* < 0.01). ^***^, Significantly different between two groups (*p* < 0.001).

To investigate whether G721-0282 targets CHI3L1 and exerts an anti-neuroinflammatory and anxiolytic effect, CHI3L1 siRNA was treated with BV-2 microglial cells to create a CHI3L1-deficient environment. The anti-neuroinflammatory effects of G721-0282 on CHI3L1-knockdown and normal cells were determined by Western blotting and qRT-PCR. In normal cells, LPS-induced elevated expressions of iNOS, COX-2, and IBA-1 were significantly reduced by G721-0282 treatment. Despite G721-0282 treatment in CHI3L1-knockdown cells, the elevated levels of iNOS, COX-2, and IBA-1 were not affected (Figure 4A), nor was the inhibitory effect of G721-0282 on activation of NF-κB signal observed in CHI3L1-knockdown cells (Figure 4B). In normal cells, the LPS-induced expression level of pro-inflammatory cytokines was significantly decreased upon treatment with G721-0282, but not in CHI3L1-knockdown cells [*n* = 8–12; TNF-α: *F*_(5, 53)_ = 32.80, *p* < 0.0001; IL-1β: *F*_(5, 50)_ = 13.93, *p* < 0.0001; IL-6: *F*_(5, 50)_ = 60.25, *p* < 0.0001] (Figure 4C). These data suggests that CHI3L1 plays a role in the inflammatory response.

### Chitinase-3-Like 1 Regulates IGFBP3-Mediated Neuroinflammation

**FIGURE 5:**
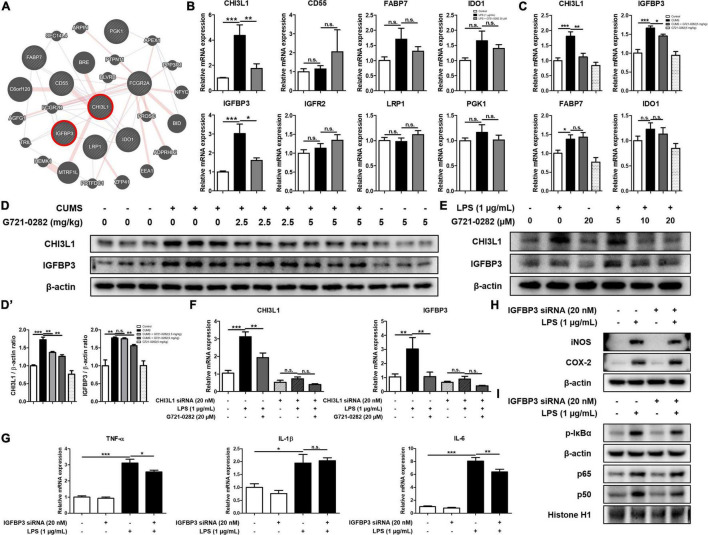
Inhibitory of G721-0282 on IGFBP3-mediated neuroinflammation. Gene network analysis associated with CHI3L1 was carried out using the web-based analysis tool **(A)**. The mRNA expression level of CHI3L1 related proteins (CD55, FABP7, IDO-1, IGFBP3, IGFR2, LRP1, and PGK1) in BV-2 cells treated with G721-0282 were assessed by qRT-PCR **(B)**. The mRNA expression level of CHI3L1 related proteins (FABP7, IDO-1, and IGFBP3) in mice brain were assessed by qRT-PCR **(C)**. The expression of CHI3L1 and IGFBP3 were detected by Western blot in the mice brain **(D)**, and their expression were normalized to β-actin **(D’)**. The loading control, β-actin, was reused in Figures 3A,B and **(D)**, because Figures 3A,B and **(D)** were obtained from the same amount of the same samples. The expression of CHI3L1 and IGFBP3 were detected by Western blot in BV-2 cells treated with siCHI3L1 and G721-0282 **(E)**. The mRNA expression level of CHI3L1 and IGFBP3 in BV-2 cells treated with siCHI3L1 and G721-0282 were assessed by qRT-PCR **(F)**. The mRNA expression level of pro-inflammatory cytokines (TNF-α, IL-1β, and IL-6) in BV-2 cells treated with siIGFBP3 were assessed by qRT-PCR **(G)**. The expression of iNOS and COX-2 were detected by Western blot in BV-2 cells treated with siIGFBP3 **(H)**. Phosphorylation of IκB, and p50 and p65 (Nuclear) translocation were detected by Western blot in BV-2 cells treated with siIGFBP3 **(I)**. *, Significantly different between two groups (*p* < 0.05). ^**^, Significantly different between two groups (*p* < 0.01). ^***^, Significantly different between two groups (*p* < 0.001).

We screened genes that were reported to be up-regulated when CHI3L1 was expressed through GWAS analysis and selected seven genes: CD55, FABP7, IDO1, IGFBP3, IGFR2, LRP1, and PGK1 [*n* = 10–11; CHI3L1: *F*_(2, 29)_ = 11.90, *p* = 0.0002; CD55: *F*_(2, 29)_ = 0.6759, *p* = 0.5165; FABP7: *F*_(2, 29)_ = 2.529, *p* = 0.1093; IDO1: *F*_(2, 29)_ = 2.794, *p* = 0.0777; IGFBP3: *F*_(2, 29)_ = 12.95, *p* < 0.0001; IGFR2: *F*_(2, 29)_ = 2.076, *p* = 0.1437; LRP1: *F*_(2, 29)_ = 1.137, *p* = 0.3429; PGK1: *F*_(2, 29)_ = 0.7190, *p* = 0.4994] (Figure 5A). We then treated BV-2 cells with LPS and G721-0282 to quantify the expression levels of eight genes including CHI3L1 by qRT-PCR [*n* = 8–9; CHI3L1: *F*_(3, 29)_ = 12.81, *p* < 0.0001; IGFBP3: *F*_(3, 29)_ = 19.41, *p* < 0.0001; FABP7: *F*_(3, 29)_ = 8.073, *p* = 0.0005; IDO1: *F*_(3, 29)_ = 2.238, *p* = 0.1050] (Figure 5B). We selected FABP7, IDO1, and IGFBP3, with the same expression patterns as CHI3L1, in the qRT-PCR data and investigated the expression levels of these three genes and CHI3L1 in the mice hippocampus (Figure 5C). In this series of studies, we found that IGFBP3 is strongly associated with CHI3L1.

We performed the Western blot to investigate the effects of G721-0282 as a CHI3L1 inhibitor on the expression level of CHI3L1 and IGFBP3 in the mouse hippocampus. The expression levels of CHI3L1 and IGFBP3 were increased in the CUMS-treated group, and the elevated expression was reduced by treatment of G721-0282 in a dose-dependent manner (Figures 5D,D’). To investigate the correlation between CHI3L1 and neuroinflammation, BV-2 cells were treated with LPS and several concentrations of G721-0282 (5, 10, 20 μM), and the levels of CHI3L1 and IGFBP3 were determined by Western blot. Expressions of CHI3L1 and IGFBP3 were increased by LPS treatment, but the increased expression was decreased by G721-0282 (Figure 5E). To determine whether CHI3L1 is a signal of IGFBP3, CHI3L1-knockdown BV-2 cells were treated with LPS and their expression levels were measured by qRT-PCR. The LPS-induced expression level of IGFBP3 in normal cells was decreased by G721-0282, but the effect of G721-0282 was not observed in CHI3L1-knockdown BV-2 cells [*n* = 6–8; CHI3L1: *F*_(5, 39)_ = 32.07, *p* < 0.0001; IGFBP3: *F*_(5, 40)_ = 6.801, *p* < 0.0001] (Figure 5F). To investigate the correlation between IGFBP3 and neuroinflammation, BV-2 cells were treated with IGFBP3 siRNA and the levels of inflammatory cytokines were determined by qRT-PCR, and related proteins and NF-κB signaling were determined by Western blotting. In normal cells, the levels of pro-inflammatory cytokines were significantly increased by LPS treatment. In IGFBP3-knockdown, BV-2 cells treated with LPS showed significantly lower expression levels of TNF-α and IL-6 than normal cells [*n* = 8; TNF-α: *F*_(3, 32)_ = 60.43, *p* < 0.0001; IL-1β: *F*_(3, 32)_ = 10.46, *p* < 0.0001; IL-6: *F*_(3, 32)_ = 114.0, *p* < 0.0001] (Figure 5G). Moreover, when IGFBP3 siRNA was treated, the LPS-induced levels of iNOS and p-IκBα and the translocation of p50 were slightly lower in the siRNA-treated group than in the control group (Figures 5H,I).

## Discussion

When we continually treated BALB/c mice with CUMS, the mice exhibited anxious behavioral characteristics, but these anxious behavioral characteristics were decreased by the administration of G721-0282. These results indicate that G721-0282, a CHI3L1 inhibitor, alleviates anxiety disorders caused by CUMS.

There have been reports showing an association between anxiety disorders and neuroinflammation. Wohleb et al. (2014) found that the expression of IL-1β and the recruitment of macrophages were increased in the mouse brain by repeated social defeat (RSD) stress, elevating anxiety-like behaviors. They also reported that the activation of microglia and anxiety-like behaviors were decreased in RSD stressed IL-1 receptor type 1 knockout mice (Wohleb et al., 2014). Uguz et al. (2009) reported that patients with rheumatoid arthritis suffer from anxiety disorders, and anxiety level was alleviated in these patients when anti-TNF-α therapy was applied. Nelson and Lenz (2017) found that mice with microglia selectively depleted by liposomal clodronate in the brain had a significantly lower pattern of anxious behavior than normal mice. These data indicate that neuroinflammation could be a significant contributing factor in the development of anxiety. In this study, we demonstrated that the mice exhibit behavioral characteristics of anxiety disorders by treatment of CUMS through a series of behavioral experiments. When G721-0282 was administered, the behavioral characteristics of anxiety disorders in mice were decreased dose-dependently. In line with previous findings showing an association between neuroinflammation and anxiety, we also found increased neuroinflammation in the hippocampus tissue of the CUMS-treated mice. Levels of inflammatory proteins such as iNOS and COX-2 and pro-inflammatory cytokines such as TNF-α, IL-1β, and IL-6 were significantly elevated in the hippocampus of CUMS-treated mice. Furtado and Katzman (2015) reported that serum levels of IL-6 and TNF-α are high in patients suffering from PTSD and OCD. It is also noteworthy that when blocking TNF-α and IL-1β signaling, the anxiety state was attenuated, suggesting that neuroinflammation is involved in the pathogenesis of anxiety disorder (Simen et al., 2006; Koo and Duman, 2009). We also found that NF-κB signal transduction, which is highly involved in neuroinflammation, was activated in CUMS-treated mice. Also, astrocytes and microglia, the representative inflammatory cells of the CNS, were highly activated in CUMS-treated mice. There have been reports that the brain-derived neurotrophic factor (BDNF) pathway can regulate several neurotransmitters, including serotonin, and thus have suggested the possibility that BDNF may have anti-anxiety effects (Martinowich et al., 2007; JiaWen et al., 2018). In our results, the level of BDNF was decreased in the hippocampus of CUMS-treated group compared that of control group, and the BDNF levels were increased in the hippocampus of G721-0282-treated groups (Supplementary Figures 3A,C). Also, we observed that the expression of BDNF was lower in the LPS-treated group and higher in the G721-0282-treated groups than that of LPS group (Supplementary Figures 3B,D). However, G721-0282 treatment significantly reduced all of these inflammatory markers, including inflammatory proteins and cytokines, markers of astrocyte and microglia, and NF-κB signaling. These results suggest that the relief of CUMS-induced anxiety is related to the anti-neuroinflammation effect of G721-0282. The dysfunction of the Hypothalamic Pituitary Adrenal (HPA) axis is closely related to the pathogenesis of psychiatric disorders including anxiety disorders (Kirsten et al., 2013). CUMS treatment increased serum CORT in BALB/c mouse, on the other hand, G721-0282 treatment decreased serum CORT in BALB/c mouse in dose-dependent manner (Supplementary Figure 4). Some studies reported results suggesting a relationship between neuroinflammation and HPA-axis. Glucocorticoids of the HPA-axis, which are increased by stress, suppress the inflammatory responses by binding glucocorticoid receptors, however, when stress develops and the continuous exposure to high levels of glucocorticoids occurs, the resistance to the anti-inflammatory effect of glucocorticoids develops and increases inflammation (Bauer and Teixeira, 2019). LPS administration causes inflammatory responses such as the production of inflammatory cytokines, which also includes inflammation of the nervous system (Ham et al., 2019a). Mayerhofer et al. (2017) have shown that LPS-treated mice showed anxiety related behaviors while at the same time increasing corticosterone. Moreover, Jang et al. (2018) reported that immobilization stress causes increased anxiety-like behaviors in mice, increased inflammation—increased activation of microglia, activation of NF-κB pathway, and TNF-α production, decreased BDNF—and at the same time increased CORT from serum. Many previous studies, including ours, have accumulated evidence that CHI3L1 is strongly associated with inflammatory responses (Steponaitis et al., 2016; Querol-Vilaseca et al., 2017; Qiu et al., 2018; Lee et al., 2019). Our previous study showed that levels of inflammatory proteins such as iNOS and COX-2 and pro-inflammatory cytokines such as TNF-α and IL-1β were lower in CHI3L1 KO mice than in WT mice in an ethanol-induced liver disease model (Lee et al., 2019). We also reported that inflammatory markers and activation of microglia and astrocytes were reduced when CHI3L1 inhibitor was administered to β-amyloid-induced neuroinflammatory mice (Choi et al., 2018). Tran et al. (2014) reported that CHI3L1 mediates activation of IL-6-mediated STAT3 phosphorylation. Gong et al. (2014) found that CHI3L1 is highly expressed in atherosclerotic patients. In CHI3L1 KO mice, the expression of TNF-α, IL-6, and MCP-1 as cardiovascular risk factors and atherosclerosis was decreased compared to WT mice (Gong et al., 2014). CUMS-treated mice showed higher expression of CHI3L1 than untreated mice. The promoter of CHI3L1 has glucocorticoid response element (GRE) sequences, and therefore it seems likely that CHI3L1 could be expressed in response to stress-related hormones. G721-0282 used in this study was one of the substances predicted to bind to CHI3L1 so as to interfere with the function of CHI3L1. Our pull-down analysis and molecular docking analysis show that G721-0282 directly binds to CHI3L1. When we knockdowned CHI3L1 on BV-2 cells, the expressions of proinflammatory cytokines and proteins were decreased and the activation of NF-κB signaling pathway was also inhibited. These data suggest that CHI3L1 is involved in anxiety through neuroinflammation and that the anti-neuroinflammatory effect of G721-0282 could be associated with the inhibition of CHI3L1-mediated anxiety.

Some studies have reported that insulin-like growth factor-binding protein 3 (IGFBP3) is upregulated when CHI3L1 is increased, suggesting that there is a connection between CHI3L1 and IGFBP3 (Steponaitis et al., 2016; Qiu et al., 2018). Using GWAS analysis and studies of gene expression patterns, we found that CHI3L1 is associated with IGFBP3. Atkins et al. (2019). reported that IGFBP3 and CHI3L1 are overexpressed in glioblastoma tumor patients and contribute significantly to the deterioration of glioblastoma. Jovcevska (2019) also reported that decreased mRNA expression levels of the CHI3L1 and IGFBP3 are associated with longer survival of glioblastoma patients. We found that IGFBP3 has the same expression pattern as CHI3L1. When CUMS was treated, CHI3L1 was upregulated in mouse hippocampus and IGFBP3 was upregulated as well. When G721-0282 was treated, the level of IGFBP3 decreased with CHI3L1. When a CHI3L1-deficient environment was established with CHI3L1 siRNA, the decrease of IGFBP3 levels by G721-0282 did not occur, suggesting that the expression of IGFBP3 is affected by CHI3L1. These results suggest that there could be a close relationship between CHI3L1 and IGFBP3. There have also been reports that IGFBP3 is increased in patients suffering from inflammatory diseases. IGFBP3 is significantly elevated in the blood of patients with rheumatoid arthritis and in patients with systemic sclerosis (Ding and Wu, 2018). Most of the functions of IGFBPs are related to the regulation of IGFs, especially since IGFBP3 has no binding preference with IGFs, so more than 90% of IGFs are associated with IGFBP3 (Ranke, 2015). Baldini et al. (2013) found that anxiety-like behavior was reduced when IGF-1 was administered to rats and that IGF-1 plays a major role in anxiety-like behavior. IGFBP3 acts as an inhibitor of IGF-1 receptor signaling by modulating binding to IGF-1 (Hu et al., 2016), and thus the anti-anxiety effect of IGFBP3 is likely to occur by inhibiting IGF-1 signaling. The role of IGFBP3 in inflammation is still controversial, and there is little research on neuroinflammation in the brain. However, there have been several studies that suggest that IGFBP3 has pro-inflammatory properties. Kielczewski et al. (2009) reported that IGFBP3 increases nitric oxide synthase activity and thus IGFBP3 increases NO generation in HUVECs. Watanabe et al. (2015) reported that IGFBP3 from reactive astrocytes leads to neuronal death by suppressing IGF-1. Although there are few studies of the direct correlation between IGFBP3 and neuroinflammation, it is well known that IGFBP3 inhibits the role of IGF-1, suggesting that IGFBP3 could play a role in inflammatory mediated anxiety. Several studies have reported that IGF-1 could inhibit neuroinflammation by inhibiting the activation of astrocytes (Labandeira-Garcia et al., 2017). Hu et al. (2018) demonstrated that IGF-1 knockdown activates the NF-κB signaling pathway, suggesting that IGFBP3, which inhibits IGF-1, may be a potential activator of NF-κB. These data indicate that the inhibitory effect of G721-0282 on IGFBP3-induced neuroinflammatory properties could be related to an inhibitory effect on the CHI3L1-mediated anxiety effect.

According to ADME prediction, G721-0282 will have high intestinal absorption rates and high BBB-penetration rates. G721-0282 does not violate either Lipinski’s rule or the Lead-like rule, indicating that its drug-likeness properties are good. G721-0282 is predicted not to exhibit acute toxicity in the cardiovascular system, lung, kidney, gastrointestinal tract, or blood as well as genotoxic, reproductive, or carcinogenic toxicities. Because of its outstanding properties and anxiolytic effects, G721-0282 can potentially be developed as a drug.

## Conclusion

The results presented in the present study suggest that by interfering with CHI3L1, it alleviated CUMS-induced neuroinflammation and, at the same time, reduced the behavioral characteristics of anxiety. Considering this, G721-0282 as the inhibitor of CHI3L1 could be used a treatment for anxiety disorders accompanying neuroinflammation.

## Data Availability Statement

The original contributions presented in the study are included in the article/Supplementary Material, further inquiries can be directed to the corresponding author/s.

## Ethics Statement

The animal study was reviewed and approved by the Institutional Animal Care and Use Committee (IACUC) of the Laboratory Animal Research Center at Chungbuk National University (CBNUR-1291-19-01).

## Author Contributions

HH designed the experiments, conducted most of the experiments, performed data analysis, generated most of the experimental mice, and was the primary writer of the manuscript. YL performed data analysis. HL contributed to preparation and performed the research. YH performed computational docking experiment. JY and SH provided advice throughout the project. JH supervised the entire project and had a major role in experimental design, data interpretation, and writing the manuscript. All authors read and approved the final manuscript.

## Conflict of Interest

The authors declare that the research was conducted in the absence of any commercial or financial relationships that could be construed as a potential conflict of interest.

## Publisher’s Note

All claims expressed in this article are solely those of the authors and do not necessarily represent those of their affiliated organizations, or those of the publisher, the editors and the reviewers. Any product that may be evaluated in this article, or claim that may be made by its manufacturer, is not guaranteed or endorsed by the publisher.

## Footnotes

1
www.genemania.org

2
http://preadmet.bmdrc.org

3
https://www.optibrium.com/stardrop/

## Abbreviations

CHI3L1: Chitinase-3-like 1
G721-0282: N-Allyl -2-[(6-butyl-1,3-dimethyl-2,4-dioxo-1,2,3,4-tetrahydropyrido[2,3-d]pyrimidin-5-yl)sulfanyl]acetamide
CUMS: Chronic unpredictable mild stress
IGFBP3: Insulin-like growth factor binding protein 3
iNOS: Inducible nitric oxide synthase
COX-2: Cyclooxygenase 2
GFAP: Glial fibrillary acidic protein
IBA-1: Ionized calcium-binding adapter molecule 1
TNF-α: Tumor necrosis factor alpha
IL-1β: Interleukin 1 beta
IL-6: Interleukin 6
NF-κB: Nuclear transcription factor-kappa B
IκBα: NF-κB inhibitor
NF-κB: Nuclear transcription factor-kappa B
EPMT: Elevated plus-maze test
OFT: Open field test
NSFT: Novelty suppressed feeding test
SPT: sucrose preference test.
