# Supplementary material for: G721-0282 Exerts Anxiolytic-Like Effects on Chronic Unpredictable Mild Stress in Mice Through Inhibition of Chitinase-3-Like 1-Mediated Neuroinflammation
Source: Front Cell Neurosci. 2022 Mar 7;16:793835. doi: 10.3389/fncel.2022.793835 (PMC8957088; doi:10.3389/fncel.2022.793835)
Supplement: Supplementary file 1 [file Data_Sheet_1.DOCX]

**Supplementary information for**

**G721-0282 exerts anxiolytic-like effects on chronic unpredictable mild stress in mice through inhibition of CHI3L1-mediated neuroinflammation**

Hyeon Joo Ham^1^, Yong Sun Lee^1^, Hee Pom Lee^1^, Young Wan Ham^2^, Jaesuk Yun^1^, Sang Bae Han^1^, and Jin Tae Hong^1^

^1^College of Pharmacy and Medical Research Center, Chungbuk National University, Osongsaengmyeong 1-ro, Osong-eup, Heungdeok-gu, Cheongju, Chungbuk, 28160, Republic of Korea

^2^Department of Chemistry, Utah Valley University, 800 W University Pkwy, Orem, UT 84058, USA

*Correspondence : Dr. Jin Tae Hong (jinthong@chungbuk.ac.kr), College of Pharmacy and Medical Research Center, Chungbuk National University, Osongsaengmyeong 1-ro, Osong-eup, Heungdeok-gu, Cheongju, Chungbuk, 28160, Republic of Korea, Tel: +82-043-261-2813, Fax: +82-043-268-2732

Hyeon Joo Ham: prodijoo0918@nate.com

Yong Sun Lee: kallintz@gmail.com

Hee Pom Lee: heepom@empas.com

Young Wan Ham: YHam@uvu.edu

Jaesuk Yun: jyun@chungbuk.ac.kr

Sang Bae Han: shan@chungbuk.ac.kr

**Supplementary Table S1. List and sequences of qPCR primers for mRNA expression**

|  | **Forward (5’→3’)** | **Reverse (5’→3’)** | **Species** |
| --- | --- | --- | --- |
| **β-actin** | GGCTGTATTCCCCTCCATCG | CCAGTTGGTAACAATGCCATGT | Mouse |
| **TNF-α** | TCTTCTCATTCCTGCTTGTGG | CACTTGGTGGTTTGCTACGA | Mouse |
| **IL-1β** | CCTTCCAGGATGAGGACATGA | TGAGTCACAGAGGATGGGCTC | Mouse |
| **IL-6** | GAGGATACCACTCCCAACAGACC | AAGTGCATCATCGTTGTTCATACA | Mouse |
| **CHI3L1** | GTACAAGCTGGTCTGCTACTTC | ATGTGCTAAGCATGTTGTCGC | Mouse |
| **CD55** | ACCTCCACTCCCAGGAAAAG | TAGAGGAGACACCGACTAGCC | Mouse |
| **FABP7** | ATGAACTTCTCCGGCAAGTACC | CTGACACCCCCTTGATGTCC | Mouse |
| **IDO1** | GCTTTGCTCTACCACATCCAC | CAGGCGCTGTAACCTGTGT | Mouse |
| **IGFBP3** | CCAGGAAACATCAGTGAGTCC | GGATGGAACTTGGAATCGGTCA | Mouse |
| **IGFR2** | GGGAAGCTGTTGACTCCAAAA | GCAGCCCATAGTGGTGTTGAA | Mouse |
| **LRP1** | ACTATGGATGCCCCTAAAACTTG | GCAATCTCTTTCACCGTCACA | Mouse |
| **PGK1** | ATGTCGCTTTCCAACAAGCTG | GCTCCATTGTCCAAGCAGAAT | Mouse |
| **BDNF** | TCATACTTCGGTTGCATGAAGG | AGACCTCTCGAACCTGCCC | Mouse |
| **CREB** | AGCAGCTCATGCAACATCATC | AGTCCTTACAGGAAGACTGAACT | Mouse |
| **TrkB** | CTGGGGCTTATGCCTGCTG | AGGCTCAGTACACCAAATCCTA | Mouse |

**Supplementary Table S2. Predicted drug likeness and toxicities of G721-0282**

| **Test** | **G721-0282** |
| --- | --- |
| Lipinski’s rule | 0 violation |
| Lead-like rule | 0 violation |
| Human intestinal absorption (HIA, %) | 100, Highly absorbed |
| Brain/Plasma equilibration rate | -2.50, Sufficient |
| Plasma protein binding | Extensively bound |
| Estrogen receptor binding | No binding |
| hERG receptor probability | Undefined |
| P-gp inhibitor probability | Undefined |
| Eye irritation | Moderate |
| Skin irritation | Moderate |
| DNA damage | Outside applicable |
| Carcinogenicity | Outside applicable |
| Chromosome aberration | Outside applicable |
| Reproductive toxicity | Negative |
| Ames mutagenicity | Negative |
| Health effects, Blood | Moderate |
| Health effects, Kidney | Moderate |
| Health effects, Cardiovascular | Moderate |
| Health effects, Liver | Moderate |
| Health effects, Gastrointestinal | Low |
| Health effects, Lungs | Moderate |

**Supplementary Table S3. Information of antibodies**

| **Name** | **Application** | **Supplier** | **Cat. No.** |
| --- | --- | --- | --- |
| CHI3L1 | WB, Pull-down assay | Abcam | ab180569 |
| iNOS | WB | Abcam | ab15323 |
| IBA-1 | WB, IHC | Abcam | ab178846 |
| GFAP | WB | Abcam | ab7260 |
| COX-2 | WB | Novus Biologicals | NB100-868 |
| NF-κB inhibitor (IκB)α | WB | Cell signaling Technology | 4814 |
| p-IκBα | WB | Cell signaling Technology | 5209 |
| p65 | WB | Cell signaling Technology | 3033 |
| p50 | WB | Cell signaling Technology | 3035 |
| IGFBP3 | WB | Abnova | PAB18394 |
| BDNF | WB | Arigo Biolaboratories | ARG57747 |
| beta-actin | WB | Santa Cruz Biotechnology | sc-47778 |
| Histone H1 | WB | Santa Cruz Biotechnology | sc-393358 |

**Supplementary Figure S1. Effect of G721-0282 on microglial activation in mice brain**

**
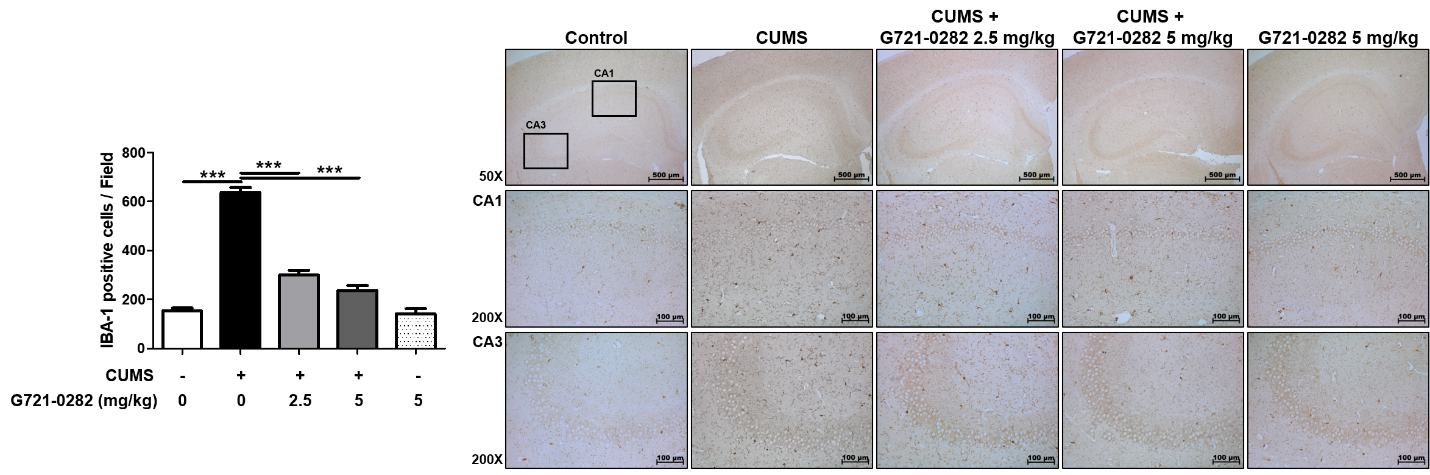
**

Immunohistochemical analysis of IBA-1 in mouse hippocampus tissue samples. IBA-1 stainings were quantified by counting the number of positive cells in the field of hippocampus.

**Supplementary Figure S2. Effect of G721-0282 on the BV-2 cell viability**


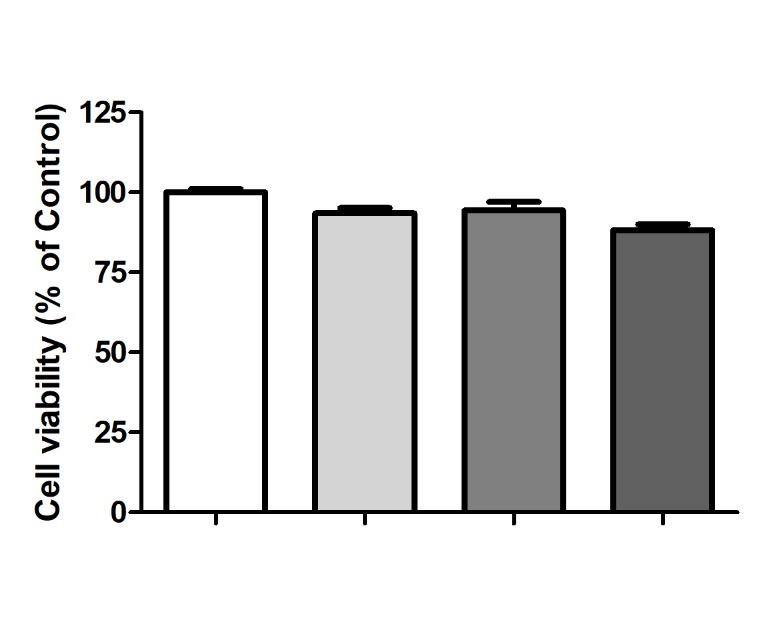


**G721-0282 (µM)**

**5**

**0**

**10**

**20**

The cell viability in BV-2 cells by G721-0282(5, 10, 20 µM) were determined by MTT assay.

**Supplementary Figure S3. Effect of G721-0282 on BDNF pathway**

**
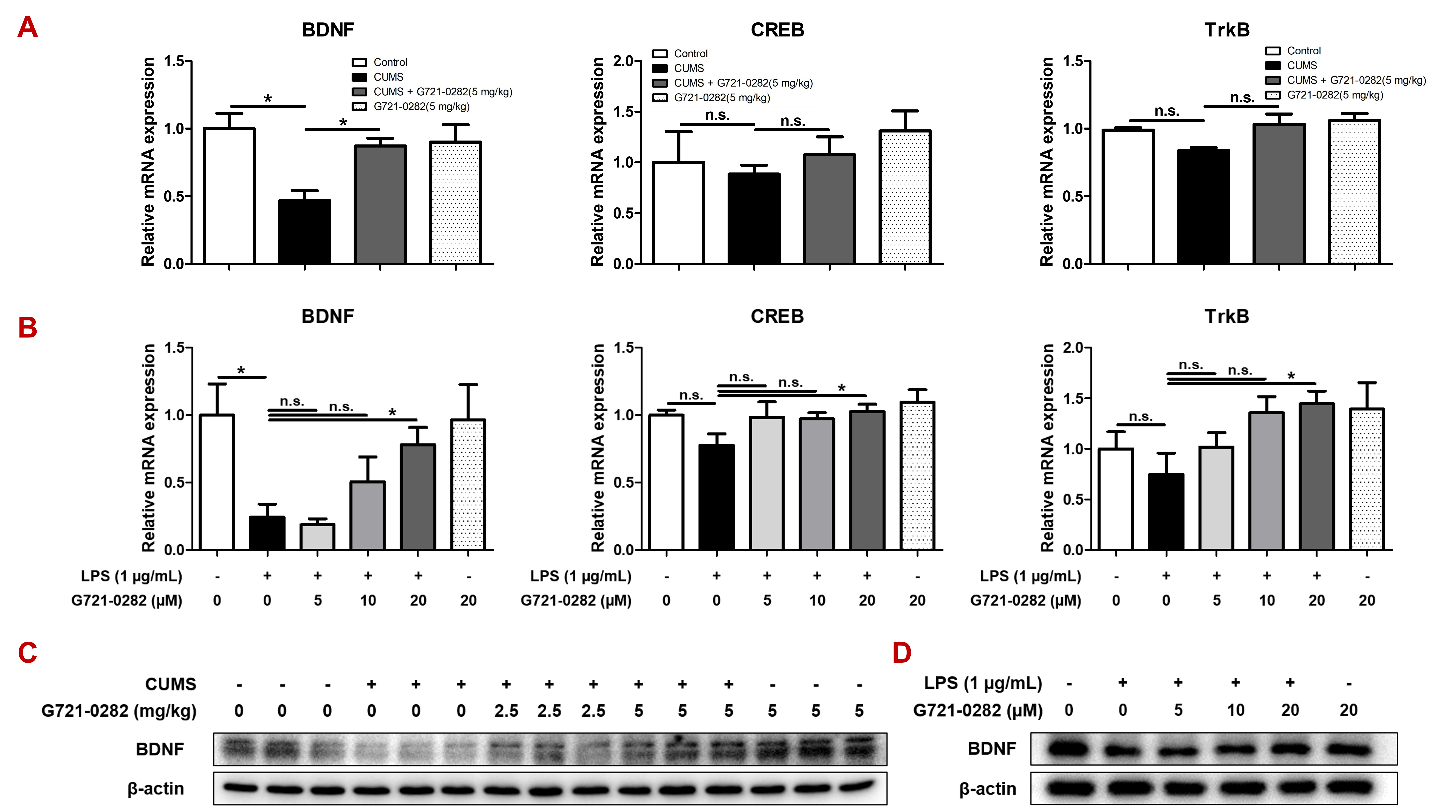
**

The mRNA expression level of BDNF, CREB, and TrkB in the mice brain hippocampus site were assessed by qRT-PCR (A). The mRNA expression level of BDNF, CREB, and TrkB in BV-2 cells treated with G721-0282 were assessed by qRT-PCR (B). Data are expressed as mean ± S.E.M. The expression of BDNF was detected by Western blotting using specific antibodies in the mice brain (C). The expression of BDNF was detected by Western blotting using specific antibodies in BV-2 cells treated with G721-0282 (D). β-actin was used as a loading control. For the cropped images, samples were run in the same gels under same experimental conditions and processed in parallel.

**Supplementary Figure S4. Effect of G721-0282 on corticosterone in CUMS-induced BALB/c mice**

**
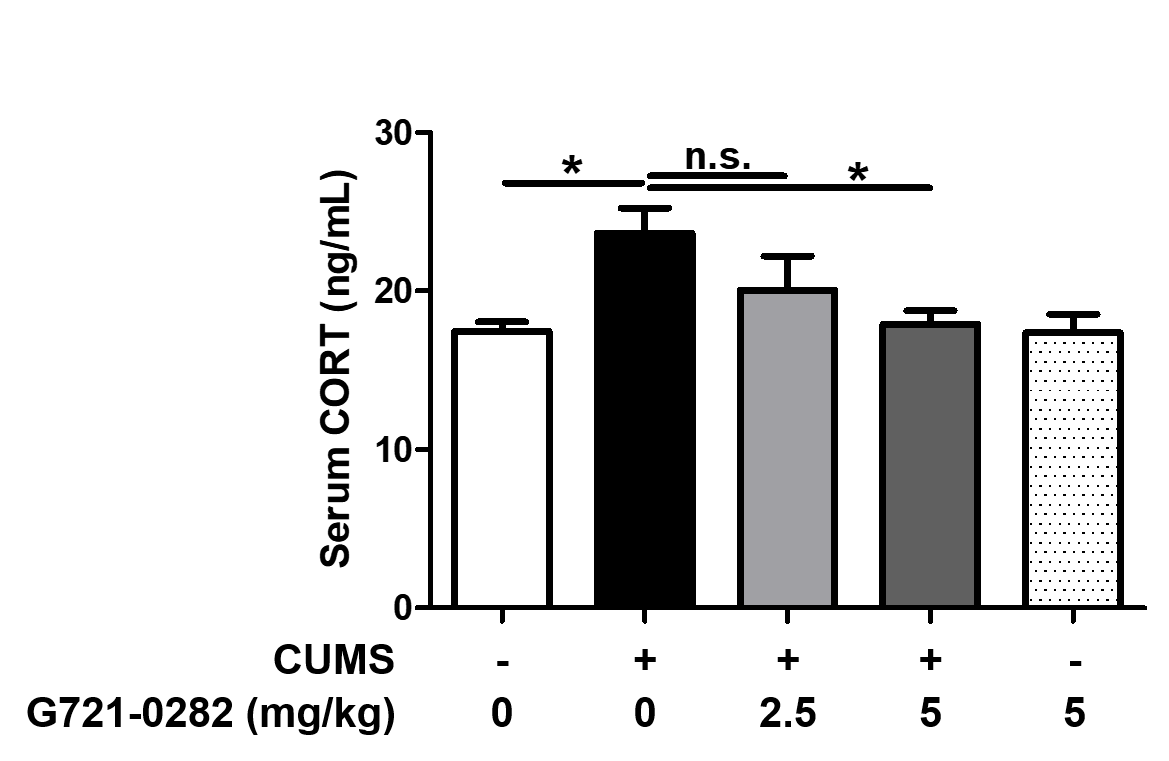
**

The serum CORT level in CUMS-induced mice were assessed using the specific ELISA kits. Data are expressed as mean ± S.E.M.
